# Microfluidics as a Novel Technique for Tuberculosis: From Diagnostics to Drug Discovery

**DOI:** 10.3390/microorganisms9112330

**Published:** 2021-11-11

**Authors:** Antonia Molloy, James Harrison, John S. McGrath, Zachary Owen, Clive Smith, Xin Liu, Xin Li, Jonathan A. G. Cox

**Affiliations:** 1 School of Life and Health Sciences, Aston University, Aston Triangle, Birmingham B4 7ET, UK; 200217293@aston.ac.uk (A.M.); j.harrison11@aston.ac.uk (J.H.); 2 Sphere Fluidics Limited, The McClintock Building, Suite 7, Granta Park, Great Abington, Cambridge CB21 6GP, UK; John.McGrath@spherefluidics.com (J.S.M.); Zach.Owen@spherefluidics.com (Z.O.); clive.smith@spherefluidics.com (C.S.); xin.liu@spherefluidics.com (X.L.); xin.li@spherefluidics.com (X.L.)

**Keywords:** tuberculosis, *Mycobacterium*, diagnostics, drug discovery, antibiotics, antimicrobial resistance, microfluidics, single-cell analysis, bioengineered models

## Abstract

Tuberculosis (TB) remains a global healthcare crisis, with an estimated 5.8 million new cases and 1.5 million deaths in 2020. TB is caused by infection with the major human pathogen *Mycobacterium tuberculosis*, which is difficult to rapidly diagnose and treat. There is an urgent need for new methods of diagnosis, sufficient in vitro models that capably mimic all physiological conditions of the infection, and high-throughput drug screening platforms. Microfluidic-based techniques provide single-cell analysis which reduces experimental time and the cost of reagents, and have been extremely useful for gaining insight into monitoring microorganisms. This review outlines the field of microfluidics and discusses the use of this novel technique so far in *M. tuberculosis* diagnostics, research methods, and drug discovery platforms. The practices of microfluidics have promising future applications for diagnosing and treating TB.

## 1. Introduction

### 1.1. Tuberculosis and Its Global Health Threat

*Mycobacterium tuberculosis* is the causative agent of the human pulmonary infection tuberculosis (TB). TB is the second leading infectious killer since the global COVID-19 pandemic. The World Health Organisation (WHO) estimates that 1.5 million people died from TB in 2020 [1]. Despite most TB strains being treatable with antibiotics, some of the key medical challenges include achieving rapid diagnostics, the rise of multidrug-resistant TB, and the poor treatment efficacy of latent TB. The current recommended treatment for drug-susceptible TB takes a minimum six-month administration of isoniazid (INH), rifampicin (RIF), pyrazinamide (PZA), and ethambutol (EMB) [2]. This first-line recommendation has failed to adapt in the last thirty-five years despite the increasing occurrence of drug resistance. Recently, a phase 3 trial provided evidence of a four-month treatment regimen with rifapentine and moxifloxacin [3]. Additionally, many efforts have been made to reduce the mycobacterial burden (reducing mortality and transmission), eradicate persistent mycobacterial populations, and to reduce drug resistance through various incentives such as END-TB [4] and WHO End TB Strategy 2016–2035 [5]. Research into the economic burden of TB has revealed a global cost of 983 bn USD from 2015–2030 if the current health status continues [6]. There is a pressing need for innovative advancements and applications which combine multidisciplinary research for combating the looming crisis of TB.

### 1.2. Mycobacterium Tuberculosis and the Pathogenesis of TB

**Figure 1:**
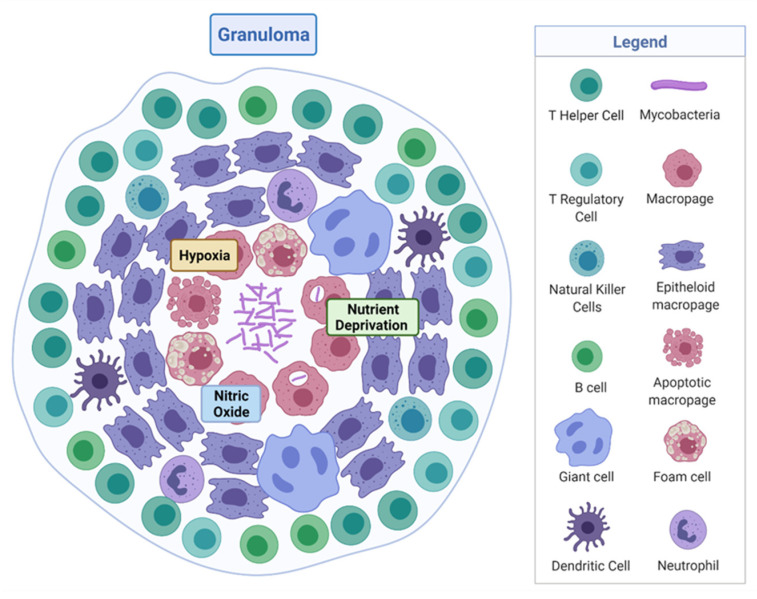
Tuberculous granuloma. Encapsulated *Mycobacterium tuberculosis* surrounded by immune cells, creating a hypoxic, nutrient-deprived, and nitric oxide environment. Adapted from “Granuloma”, by BioRender.com (2020). Retrieved from https://app.biorender.com/biorender-templates, accessed on 27 September 2021.

*M. tuberculosis* is a rod-shaped acid-fast-staining bacterium of the Actinomycete family [7]. The unique “waxy” cell envelope of *M. tuberculosis* contains a core composed of peptidoglycan and the highly branched polysaccharide arabinogalactan. This is covalently attached to the unique mycolic acids that cover the bacteria with a mycobacterial outer membrane which allows cellular integrity and virulence [8]. This self-protection permits the organism to evade the host immune system and prevents antibiotic penetration [8,9]. The molecular pathology by which *M. tuberculosis* evades the host and causes disease is complex, involving a dynamic range of immune cells. The organism infects the host after the inhalation of droplet nuclei spread by aerosolisation from an infected individual, which then resides in the respiratory tract [10]. There are various types of infection that can manifest from *M. tuberculosis* in individuals—one where the infection clears, one with an active infection treated with a course of antibiotics, and one which remains in a latent form [11]. Upon infection, the early innate immune system emerges with an influx of neutrophils, monocytes, macrophages, and dendritic cells of the lungs [12]. Through phagocytosis, bacteria are consumed by alveolar macrophages to form a phagosome and then subsequently eliminated through the formation of phagolysosomes [13]. However, *M. tuberculosis* can avoid this host defence response by persisting in phagosomes and inhibiting lysosome fusion [13]. The subsequent established intracellular infection and influx of immune cells which surround the site of infection forms a tuberculous granuloma [14]. The early granuloma (Figure 1) consists of the infected macrophages in the centre, enclosed by foamy macrophages and other mononucleated cells, and surrounded by lymphocytes [15]. During the maturation of the granuloma, a fibrous capsule encloses the macrophage centre and eventually forms necrotic lesions, leading to caseation [14,15]. 

Here, *M. tuberculosis* can survive in a dormancy state known as non-replicating persistence (NRP). The external pressures such as hypoxia, nutrient deprivation, low pH, and high CO_2_ created by the hostile host environment induce this survival response of the bacteria [16]. The NRP state can relapse into active disease, especially in high-risk groups such as immunodeficient individuals, persons infected with human immunodeficiency virus or undergoing organ and haematologic transplantations [11]. Houben and Dodd (2016) previously estimated that NRP TB infected approximately 1.7 billion people in 2014 by generating an annual risk model of infection between 1934 and 2014 [17]. The issue posed by the ability of NRP *M. tuberculosis* to effectively hide within the hostile environment of the granuloma is that not only does the immune system keep the bacteria trapped, it also physically restricts penetration by antimicrobials, thus protecting *M. tuberculosis* from antibiotic activity.

### 1.3. Current Diagnostics, Research Methods, and Treatment

Early diagnosis and accurate detection of TB infection is essential for effective treatment options, especially in low-income and high-burden countries. Conventional TB diagnostics include microscopy (Ziehl–Neelsen staining), which provides 22–43% low sensitivity for a single smear [18]. Other methods include chest radiography, which is limited in resource-constraint locations [19], and liquid/solid culturing, which requires suitable levels of biosafety [20]. Diagnosis of latent infection requires a tuberculin skin test or interferon-gamma release assays. However, both of these tests do not identify individuals that will progress to active disease [20]. 

The phenotypic evaluation of clinical isolates, by culturing *M. tuberculosis* in the laboratory in the presence of different concentrations of antimicrobials, is traditionally used to detect drug-resistant strains. The turnover time for these results is extensive, by which point the patient’s health will have deteriorated [21]. Improvements in molecular diagnostic testing have revolutionised detection, such as the genotypic test Cepheid GeneXpert MTB/RIF, which can give a readout in two hours of TB detection and RIF resistance [22]. Additionally, whole-genome sequencing of TB is expanding with support from the WHO but still relies on culturing samples for weeks and technical methods in preparing genomic DNA for sequencing [23]. User-friendly and non-laborious detection methods, which are portable, are required to improve detection time at lower cost. 

Experimental modelling of TB has historically helped scientists to discover the pathogenicity, physiology, metabolism, and genetic make-up of the organism. Challenges arising for researchers studying mycobacteria are the characteristics of slow growth rate, hydrophobic aggregation of cells in the absence of non-ionic surfactant when grown in culture, and the need for the containment of aerosols which brings additional safety precautions, including a biosafety level 3 (BSL-3) facility [7]. Additionally, the investigation of heterogenicity is difficult in bulk cultures compared with single-cell analysis [24]. Animal models are abundant for studying TB, such as zebrafish, rabbits, guinea pigs, and mouse models [25]. However, absent is the ability of each model to represent all aspects of the physiological state of the cell and tissue environment [25], or they lack the lung immune system entirely [26]. There have been extensive reviews detailing the methods used to experimentally model this organism in its non-replicating state [27,28,29]. However, to date, no NRP models mimic all the physiological features of the bacteria in this condition. Therefore, novel in vitro experimental models of TB are imperative. 

Research groups often use variable types of nutrient media, inoculum starting points, and reading endpoints, making the standardisation of antimicrobial testing for *M. tuberculosis* difficult. Efforts have been made to standardise testing; however, protocols are still time consuming [30,31]. TB has shown resistance to antimicrobials, including multidrug-resistant strains resistant to RIF and INH [1]. Worryingly, extensively drug-resistant TB is increasing, which is resistant to RIF, INH, Fluoroquinolone, and Kanamycin [32]. There is an urgent need for shorter and more effective treatment regimens, as well as the discovery of novel compounds. Biomedical engineering approaches such as applied technology have advanced the field of drug discovery and will continue to develop new research models with ever more accurate mimicry of human physiology. 

## 2. Microfluidics

### 2.1. Technological Advancement of Microfluidics

We require innovative and advanced technology to advance *M. tuberculosis* research, such as new high-throughput methods of phenotypic assessment. Advancements in microtechnology, particularly at the micro and nanoscale, have had wide microbiological applications. Microfluidics is a rapidly growing field which comprises multidisciplinary expertise in biology, chemistry, physics, and engineering. A simple definition of microfluidics is the systematic manipulation of systems that have microscale channels where fluid volumes of nanolitres to attolitres can flow in geometric configurations [33]. Well suited to the scale of bacteria, microfluidics can produce biological assays in parallel with well-defined, controllable environmental conditions. Advantageously, the methodological approach of manipulating fluids opens a pathway to reduce animal models. This review will outline the field of microfluidics, and discuss the recent use of microfluidic techniques in TB diagnostics and drug discovery. 

### 2.2. The Physics of Microfluidics

Different physical forces direct the behaviour of a fluid in a system. The essential behaviour of a hydrodynamic system and the dominant physical effects are typically analysed by characteristic, dimensionless numbers. These numbers compare the relative importance of competing forces or may be alternatively described as ratios of characteristic length, time, or energy scales. The most prominent number in microfluidics is the Reynolds number, describing the ratio of inertial forces and viscous forces [34]:

(1) Re=ρvLμ

with mass density (the ratio of a mass of fluid to its volume of the fluid kg/m^3^) ρ, velocity v, dynamic viscosity μ, and a characteristic length L describing the dimensions of the system 

Due to the capacity of microfluidic systems and the corresponding slow flow velocities, the value of the number is typically Re<1, causing laminar flow, a regime also referred to as Stokes flow, which is a subtype of laminar flow. Mathematically, this regime is governed by the Stokes equation when Re<1 [34]:

(2) ∇p+f=μΔv

which is a linearization of the Navier–Stokes equation whereby the inertia term $\rho \left(\frac{\partial v}{\partial t}\right)+v\;\nabla v$ has been neglected. This inertia term represents the fluid version of the acceleration part $m\frac{\mathrm{dv}}{\mathrm{dt}}$ in Newton’s second law vanishing for small Reynolds numbers. The stationary Stokes equation as shown here in Equation (2) relates the gradient of the pressure p to the change in velocity v and an external body force f (e.g., a gravitation or dielectrophoretic force), with ∇ and Δ being the Nabla and Laplace operator, respectively. In other words, the pressure gradient and the external body force drive the fluid flow. However, for some very high-throughput applications operating at high flow velocities, the assumption of small Re does not necessarily hold true as the regime of “inertia microfluidics” is entered. In this regime, the full Navier–Stokes equation including its non-linear inertia term must be considered. 

With the absence of turbulent flow, the mixing of parallel, laminar fluid flows in microfluidics only occurs by diffusion, which can be a slow process. The Péclet number (Pe) describes the ratio of the rates of convection and diffusion for suspended objects, and is given by [35]:

(3) $$\mathrm{Pe}\equiv \frac{\mathrm{vw}}{D}=\frac{\mathrm{diffusion}\;\mathrm{time}}{\mathrm{convection}\;\mathrm{time}}$$

where v and w are the flow velocity and microchannel width. The diffusion coefficient is given by D and the following Stokes–Einstein relation enables the calculation of D for spherical objects:

(4) $$D=\frac{\mathrm{kT}}{6\pi \mu a}$$

In Equation (4), k is the Boltzmann constant, T is the absolute temperature, and a is the hydrodynamic radius of the suspended object. For micrometre-sized objects, the effect of diffusion is generally very small and does not greatly influence overall particle trajectory. However, as object size decreases, diffusivity increases, meaning that separation efficiency will be decreased unless flow velocity is increased. 

Where mixing is desired, passive mixing can be introduced when designing channel geometries, such as ridges, network gradient generators, and vortex micromixers. Alternatively, active mixing can be introduced by external energy, for example, electrokinetic forces and thermal actuation [36]. Active and statistical mass transport can occur in microfluidic systems [35].

As the geometrical dimensions of a microchannel decrease, the fluidic resistance increases because of friction between the microchannel walls and the body of fluid. Generally, the surface area to volume ratio becomes larger as the channel geometry becomes more complex, and so does the fluidic resistance (R), which can limit the fluid flow rate (Q). For pressure-driven flow, the relationship between these properties is given by:

(5) $$Q=\frac{\Delta p}{R}$$

where Δp is the pressure difference along the microchannel—an increasing R value would cause a continuing decrease in Q.

The three-dimensional shape of the channel governs the method required to estimate the fluidic resistance of the microchannel. In a high aspect ratio rectangular microchannel, whereby channel width or height (h) are larger than the other dimension, the fluidic resistance is given by [37]:

(6) $$R=\frac{12\mu l}{\mathrm{wh}³}$$

where the channel length is l. On the other hand, in a low aspect ratio rectangular microchannel (w≈h), the resistance is given by [37]:

(7) $$R=\frac{12\mu l}{\mathrm{wh}³}{\left[1-\frac{h}{w}\left(\frac{192}{\pi^{5}}\overset{\infty }{\underset{n=1,3,5}{\sum }}\frac{1}{n^{5}}\tan h\left(\frac{n\pi w}{2h}\right)\right)\right]}^{-1}$$

The resistance in a microchannel with a circular cross-section can be calculated using:

(8) $$R=\frac{8\mu l}{\pi r⁴}$$

where r is the radius of the circular cross-section.

### 2.3. Droplet Microfluidics

**Figure 2:**
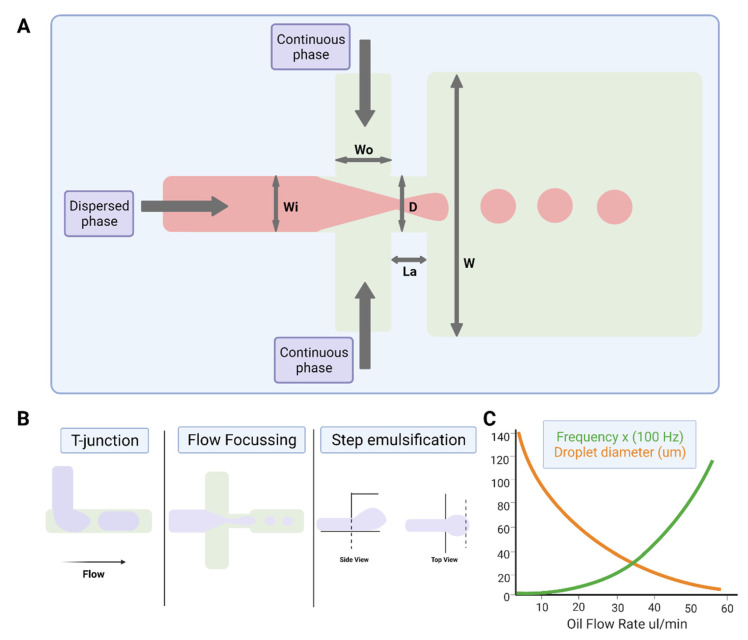
Water-in-oil droplet-generation microfluidics. (**A**) Production of water-in-oil droplets using a flow-focusing design. The dispersed phase is squeezed by two counter-streaming flows of the carrier phase, forcing drops to form and detach. (**B**) Droplet generation using T-junction, flow-focusing geometry and step emulsification. (**C**) Graph showing that droplet size decreases, and frequency of formation increases with increasing oil flow rate. Figures created on Biorender.com, accessed on 9 November 2021.

The study of multiphase flows, often termed droplet microfluidics, is a subset of the microfluidics field in which nano- to femtoliter volume droplets can be routinely generated by drop-making micronozzles in a carrier fluid at production rates exceeding 10 kHz (Figure 2). Recently, droplet production rates exceeding 1 MHz have been reported [38]. High droplet production rates enable the possibility of undertaking millions of individual experiments within a single microfluidic device. Further, droplet microfluidic systems enable the efficient control of droplet volumes, repeatable and reliable droplet manipulation, high-throughput capability, single-cell analysis capabilities, and can be fully automated. Their applications include chemical and biological assays [39,40], inorganic chemistry [41], and protein crystallisation [42,43]. The reader is referred to recent reviews on various assays, screens, and studies enabled by droplet microfluidics [44], as well as its applications in drug discovery, transcriptomics, and molecular genetics [45].

#### 2.3.1. Physics of Droplet Microfluidics

For single-cell analysis applications using droplet microfluidics, liquid/liquid emulsions comprising a cell-friendly aqueous interior, and a surfactant-stabilised fluorous oil are often used. Inclusion of cells in the aqueous, dispersed phase, results in the encapsulation of individual cells within the emulsion. The droplet occupancy number can be controlled by altering the concentration of cells within the dispersed phase and calculated using Poisson statistics [46]. To enable such encapsulation, the two immiscible fluids are typically flowing and converge within droplet microfluidic systems such that they are separated only by their interfaces (Figure 2), giving rise to interfacial tension $\gamma_{i}$ between the two fluids. The term “surfactant” is a shortening of the term “surface active agent”, and describes an amphiphilic molecule, i.e., with different groups having affinities for different immiscible phases (water/oil, water/air, oil/air). In droplet microfluidics, surfactants have a basic role: to guarantee that droplets do not coalesce, which is the minimal requirement for the use of droplets as microreactors. This amphiphilic property drives surfactant molecules to the interface of the two fluids: the surface tension of the interfacial layer and interfacial tension between the two phases is decreased. The decrease in surface tension is directly influenced by the amount of molecules adsorbed at the interface, as given by the Gibbs adsorption isotherm for dilute solutions [47]:

(9) $$\Gamma =-\frac{c}{\bar{R}T}\frac{d\gamma }{dc}$$

where Γ is the surface concentration, c the surfactant bulk concentration, T is temperature, $\bar{R}$ the gas constant, and γ the surface tension.

As surfactant adsorbs to the interface, the interface rigidifies: the loss of mobility imposes a change in the boundary condition at the interface which slows it down. The origin of the rigidification is the so-called Marangoni effect: as a drop moves, the surfactant distribution is non-uniform, with an excess at the rear of the drop [48]. The non-uniform surface concentration leads to a gradient in surface tension (the surface tension is decreased at the drop rear) which generates a stress opposed to the flow. When surface tension exists at the interfacial layer of two phases, with surfactant added to the oil phase, the Marangoni flow counteracts film drainage to counteract phase mixing, which limits coalescence in droplet systems. 

In conjunction with the interfacial tension between the two phases, complex phenomena arise that are governed by various dimensionless numbers containing the surface tension. The balance of inertial, viscous, and interfacial tension forces govern droplet formation and subsequent droplet flow. The relationship between the inertial and interfacial tension forces of the aqueous phase is quantified by the Weber number [34]:

(10) We=ρLv2γ

which is often paired with the Capillary number [34]:

(11) Ca=μvγ

when determining droplet formation dynamics. Ca describes the ratio of viscous to interfacial forces and plays an important role in the characterisation of two-phase flows. Meanwhile, another dimensionless number, the Ohnesorge number [49]:

(12) $$Oh=\sqrt{We}/Re=\mu /\sqrt{\rho \gamma L}$$

describes the relationship between the inertial, viscous, and surface tension forces on droplet microfluidic flow.

Numerous biomedical applications require materials such as solids or gels, and not liquids [50]. Solid particles made from polymeric and biological materials are used in drug delivery [51,52,53,54] and hydrogels [55] and are being studied for the encapsulation of cells in drug studies or for implantation. Many droplet microfluidic systems have been created to generate solid particles as well as hydrogel beads using various approaches [56,57,58]. Dissolved polymers add an elastic component to the fluid that further enriches flow behaviour. The Weissenberg, Deborah, and Elasticity numbers, Wi, De, and El, describe elastic effects within microfluidic flows due to the presence of deformable materials such as polymers [34]. The Weissenberg number, $Wi=\tau_{p}\dot{e}$ or $\tau_{p}\dot{\gamma }$, relates the polymer relaxation time to the flow deformation time, in the form of either the inverse extension rate ${\dot{e}}^{-1}$ or shear rate ${\dot{\gamma }}^{-1}$. When Wi is large, i.e., approaching 1, the polymer does not have sufficient time to relax and is deformed significantly. When Wi is small, the polymer has sufficient time to relax before the flow deforms it significantly, while perturbations to equilibrium are small.

Another relevant time scale $\tau_{flow}$ characteristic of the flow geometry may also exist in droplet microdluidic systems. For example, a channel that contracts over a length l introduces a geometric time scale $\tau_{flow}=l/v$ which is required for a polymer to travel through the channel. Likewise, an oscillatory flow introduces an oscillation time, where the flow time scale $\tau_{flow}$ can be long or short compared with the polymer relaxation time $\tau_{p}$, resulting in a dimensionless ratio known as the Deborah number $De=\tau_{p}/\tau_{flow}$. For both Wi and De, the equations do not directly depend on γ but are introduced due to the deformation of objects enclosed by an interface. The polymer relaxation time depends on γ, however.

As the flow velocity increases, elastic effects become more influential and Wi and De increase. However, the Reynolds number Re increases too, meaning that inertial effects can also become more influential. The Elasticity number [34] $El=\frac{De}{Re}=\tau_{p}\mu /\rho h^{2},$ where h is the shortest dimension regulating the shear rate, indicates the relative importance of elastic to inertial effects. Significantly, El is independent of flow rate and depends only on the geometry and material properties of the fluid.

#### 2.3.2. Device Geometries for Droplet Production

The most common channel geometries used for microdroplet generation include the T-junction, flow-focusing, and co-flow nozzles, and step-emulsification devices (Figure 2), each with their own benefits and shortfalls [59,60].

Droplets can be produced hydrodynamically within a T-junction system in the squeezing, dripping, or jetting regimes, whereby $Ca_{squeezing}<Ca_{dripping}<Ca_{jetting}$ [59]. Using the case of water-in-oil droplet systems as an example, constriction of the oil phase causes droplet termination/production, i.e., when the aqueous drop fills the geometric nozzle and causes resistance by pinching the oil flow.

A flow-focusing junction comprises two immiscible phases converging at a cross junction. The dispersed phase flows towards the junction in a single channel, and the continuous phase flows towards the junction in two diametrically opposed channels, each perpendicular to the dispersed phase (Figure 2). The dispersed phase is pinched off by the two incoming streams of the continuous phase, resulting in the generation of droplets at the drop-producing nozzle. Different nozzle dimensions influence the range of droplet volumes possible. These resulting droplets flow away from the junction through a channel opposite the incoming dispersed phase. By varying the flow rates of each phase, different sizes of droplets can be created. Whilst more complex than T-junctions, flow-focusing junctions offer more monodispersed and controllable droplet formation.

Co-flow droplet generators were first described by Cramer et al. [61], and utilise a thin capillary streaming the dispersed phase into a channel surrounded on two sides (Quasi-2D) or all sides (3D) [62] by the continuous phase. Quasi-2D junctions are often made using traditional soft lithography techniques [63], whilst 3D junctions are made by inserting a tapering glass capillary into a rectangular channel [64]. 

Step emulsification generators [65] (Figure 2) create droplets by altering the channel geometry to induce a rapid change in capillary pressure which drives the formation of droplets. The change in capillary pressure results from a step within the channel which causes a stream of the dispersed phase to “fall” off a step into the continuous phase. Step emulsification has benefits over other droplet-formation methods as it can be easily and massively parallelised. Despite this, the method has some disadvantages, for example, it is more sensitive to obstructions at the nozzles, which can affect droplet monodispersity [66]. 

#### 2.3.3. Active Drop Formation

Using an external input of energy can also dictate droplet generation, termed “active droplet generation” application of an external force can drive the creation of droplets. There are many techniques for active droplet formation including electrical [67,68], magnetic [69], centrifugal [70], optical [71], thermal [72], piezo-electrical [73], and surface acoustic waves [74]. Active generation methods often require more complex instrumentation setups and are therefore typically more expensive, and less accessible. Active droplet generation designs have enabled the regulation of one or more parameters such as droplet volume [75,76], generation rate [77], and also on/off switching capabilities [74,78], e.g., making it possible to produce droplets one at a time as and when required.

#### 2.3.4. Droplet Sensing and Manipulation

Droplet sensing is important for the identification and/or manipulation of droplets, and for the automation of sequential droplet activities in microfluidic Lab-on-Chip devices and/or instruments. When performing time-dependent tasks such as the manipulation of specific droplets at a specific on-chip location, droplet sensing is crucial to ensure triggered actions have the correct timing. Further, as the number of manipulation events increases, the management and automation of droplet manipulation activities needs precise, reliable information about the location, size, frequency, velocity, and/or content of droplets at certain locations within the system [79]. Two frequently utilized methods of sensing droplets in closed microfluidic channels are optical [80,81,82] and electrical [83,84,85] detection, for which the reader is referred to expert reviews [85,86,87]. To sense the interior contents of droplets, techniques such as capillary electrophoresis [85], mass spectrometry [88,89], and Raman spectroscopy [90] have been used in microfluidics, and the reader is also directed to specialised reviews [87,91] on this topic. 

The efficient manipulation of droplets [92], i.e., to perform activities such as droplet splitting, trapping, fusion, sorting, and/or to manipulate the interior droplet contents, is important in a range of research and industrial applications across various disciplines, such as biotechnology, molecular biology and analytical chemistry. Individual droplets can be manipulated in flow via a variety of techniques, e.g., passively and hydrodynamically upon careful geometrical design, or, alternatively, using active forces [36]. Many physical approaches from magnetic [93,94] to electrophoretic [95], dielectrophoretic [96,97], optic [98,99,100], pneumatic [101] and acoustophoretic [102,103,104] have been used to manipulate droplets in a microchannel—the reader is encouraged to visit the prescribed references, where a technical understanding of some of the various methods described in the literature can be gained.

### 2.4. Microfluidic Chip Materials and Microfabrication

Some of the most frequently used materials in microfluidics include thermoplastics, polydimethylsiloxane (PDMS), inorganic materials such as glass or silicon, paper, and even devices made by 3D printing, a newer approach to fabrication [105]. The most frequently used techniques for manufacturing microfluidic devices include micromachining, soft lithography, embossing, in situ construction, injection molding, and laser ablation—the reader is referred to expert reviews on such methods [35,37,105,106,107]. The most suitable method of device fabrication and material selection often depends on the specific application of the device. For example, a prerequisite for microfluidic devices to be used in biological investigations is that they must of course be biocompatible. Further, chips to be used for biological applications should be manufactured in a clean room setting to prevent the micro-channel being contaminated by dust or other matter [35]. Thermoplastics and PDMS are often selected as the material of choice as they are well researched and microfluidic chip fabrication with these materials is generally lower cost than glass or silicon [108,109,110]. Paper microfluidics have extremely low cost and can be used to measure desired molecules quickly by visual inspection [111,112,113,114].

Silicon micromachining was firstly developed for application within the field of microelectromechanical systems (MEMS) but was subsequently one of the first techniques to be used for the microfabrication of microfluidic systems [115]. The well-understood surface modification properties of silicon, plus the material’s considerable chemical resistance and ease of design, make silicon a seemingly desirable material for creating microfluidic devices for biological applications [115]. Despite that, silicon devices are not transparent to visible light, which means that such devices are not well suited for fluorescence-based detection or imaging applications [116]. However, making a composite device consisting of transparent materials such as glass or polymers, which enclose silicon microchannels, can improve suitability for imaging and fluorescence-based activities [116].

Glass has excellent analysis performance due to its biocompatibility, optical transparency, low fluorescence background, surface stability, and chemical resistance [116]. However, glass fabrication processes are generally complex, sometimes involving etching using hazardous substances such as hydrofluoric acid and/or femtosecond laser-based fabrication procedures [117] which require a high degree of training and safety precautions. Furthermore, high temperature, often in combination with high pressure, is typically required during bonding. This means that dedicated equipment is often required for fabrication, and that glass devices suffer from complications in preloading reagents before assembly, which can be problematic for some biological applications [105]. 

Soft lithography is one of many techniques used to fabricate microfluidic chips, which has largely driven the use of PDMS as a commonly used microfluidic device material. By contact printing, replica modelling and embossing, soft lithography can be used to create micro-patterns [118]. The procedure includes making a master mould containing a design made by computer-aided design (CAD). PDMS and a crosslinking agent is poured on top of the mould and placed in a high-temperature incubator. Once hardened, it is peeled from the mould to obtain a replica of the master. Access holes are punched for inlet and outlet tubes and the PDMS is placed on a glass slide and bonded by plasma treatment [119]. 

Thermoplastics have been extensively researched, refined and used for the mass production of high-quality goods, since their initial industrial uses in the 1930s [120]. Various thermoplastics exist that have been used in microfluidics, including cyclo olefin (co) polymer (COC/COP), polymethyl methacrylate (PMMA), polystyrene, polytetrafluoroethylene (PTFE), and polyetheretherketone (PEEK)—an excellent review by Gencturk et al. [110] evaluates the physical properties of thermoplastics used in microfluidics, and the present state of the development and applications of thermoplastic microfluidic systems used in cell biology and analyses. PMMA is used as an example, which is widely used in research laboratories because it is optically transparent and can be manipulated with fabrication methods such as hot embossing, laser ablation, or precision milling [110]. This material is useful for small-scale prototyping/production [121]; however, the variability inherent in PMMA devices made by these fabrication methods often makes them unsuitable for large-scale commercial production. For example, channel smoothness can be low, and the heated sealing process can cause deformations which give variability between devices. COP/COC is generally a better material choice than PMMA due to its biocompatibility, favourable optical properties, low water uptake, low binding affinity for proteins, rigidity, strength, and stability [122,123,124,125]. Furthermore, COC has excellent moldability, making it a good material for microfabrication by hot embossing [106].

The use and prevalence of paper-based microfluidics has increased significantly in recent years due to the compatibility of such devices in point-of-care or point of-use testing applications, plus their simplicity, fundamental low cost, biocompatibility, and hydrophilicity [114,126]. Various medical conditions (e.g., pregnancy testing, virus assays, etc.) can be identified/evaluated using paper microfluidic systems [114]. Fluid flow in paper devices does not require a driving external force and can instead rely on capillary force to drive fluid flow, which is caused by the intermolecular force between the fluid and the porous cellulose matrix of the material [127]. Paper-based diagnostic devices are simple to use, disposable, low cost, and environmentally friendly [128]. The disposable nature of paper and paper-derived materials reduces the risk of cross contamination, and the low cost of these materials allows broader application and more frequent testing.

An emerging microfabrication method which may overcome the limitations of prior microfluidics fabrication techniques is 3D printing, which enables the prototyping of devices at lower cost and fabrication time compared to techniques such as soft lithography or hot embossing [105]. Furthermore, complex 3D structures can be manufactured, without the need for a cleanroom environment. Three main 3D printing technologies exist: fused deposition modelling, PolyJet, and stereolithography. Each technology has advantages and disadvantages—the reader is directed to a specialist review to understand each of these methods [105].

## 3. Recent Application of Microfluidics for TB

### 3.1. Applications of Microfluidics for Diagnostics and Detecting Drug-Resistant Strains

Employing microfluidics is a promising approach for rapid and cost-effective diagnostics for *M. tuberculosis*. Detecting the pathogen with robust and reproducible fluidic models offers capabilities for clinical procedures and scientific exploration. Interestingly, a bacteria enrichment microfluidic chip and a microfluidic immunoassay chip have detected airborne *M. tuberculosis*. Jing and colleagues (2014) validated a method whereby a micro-pump draws air containing bacteria into the enrichment fluidic chip and then a full immunoassay reaction is performed on a separate chip. The method offers the potential to accurately screen *M. tuberculosis* in the aerosol [129]. Airborne *M. tuberculosis* currently requires long cultivation due to the low concentration in air samples. Capturing and directly detecting airborne *M. tuberculosis* will aid effective disease prevention and control as there is a requirement to detect samples directly from patients for quicker analysis. The small volume sizes in microfluidic chip cultivation provides rapid detection at lower sample concentrations. Diagnosing TB, especially in developing countries, requires low-cost point-of-care technologies. A paper-based microfluidics system detected sputum samples containing mycobacteria. The system used enabled the decontamination of non-mycobacteria and storage of the sputum sample [130]. A laser-etched indium tin oxide glass and PDMS microfluidic chip were used to rapidly detect and quantitate *M. tuberculosis* with high sensitivity within forty-five minutes. By creating an eight-chamber microfluidic electrochemical system with real-time loop-mediated isothermal amplification (LAMP), amplification of three respiratory related infections including *M. tuberculosis* could be monitored by measuring the electrochemical signal of methylene blue [131]. Here, a microfluidic chip, with different sample chambers, provides cost- and time-efficient detection which would benefit clinicians to decide on optimal antibiotic treatments.

Six species of mycobacteria, including nontuberculous species and members of the *M. tuberculosis* complex, were detected by combining a closed system of bead-beating, droplet fluidics, and surface-enhanced Raman spectroscopy. The spectral information obtained from the vibrational signals of the mycobacterial cell wall component, mycolic acid, effectively identified the different species. This is a promising step forward for ensuring the correct treatments are administered for the correct infections [132]. Small channel dimensions enable the manipulation of cell environments and thus can represent improved biological investigation. A potential method for quantitively detecting *M. tuberculosis* in droplet microfluidics was developed by detecting cells that express the endogenous β-lactamase, BlaC—an enzyme marker naturally expressed by *M. tuberculosis*. By encapsulating a specific fluorescent probe of BlaC and samples of bacterial strains that express BlaC in droplets, the researchers could calculate the initial concentration of cells based on fluorescence [133].

Other researchers have combined PCR techniques with microfluidics. Ip et al. (2018) used a single chip comprising positive and negative reaction chambers, as well as small liquid handling chambers. They performed isolation of *M. tuberculosis* H37Ra with magnetic beads and differentiation of live/dead bacteria with propidium monoazide dye, followed by RT-PCR and optical detection within two hours. By measuring the threshold cycle number, a low detection limit of 14 colony-forming units per reaction was achieved [134]. Besides the above new PCR microfluidic approaches, genetically detecting *M. tuberculosis* without the laborious need for PCR amplification has been achieved. For example, Domínguez et al. (2015) created a micro-cantilever platform, where hydration-induced stress could identify *M. tuberculosis* and RIF resistance within 1.5 h [135]. 

Previously, label-free DNA of *M. tuberculosis* from clinical isolates was detected by an integrated system of microfluidics and electrochemical biosensing. The platform consisting of a monolithic chip and multiwall carbon nanotubes detects *M. tuberculosis* without the need for DNA amplification [136]. Another biosensing device was developed to detect MPT64—an antigen secreted by *M. tuberculosis.* The protein is a biomarker for actively dividing mycobacteria, detected by electrochemical impedance spectroscopy and synthetic aptamers integrated with a microfluidic chamber [137]. 

**Figure 3:**
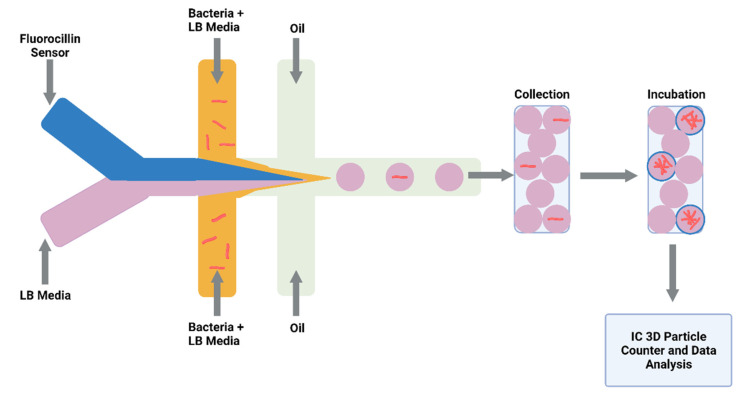
Schematic illustration of droplet system coupled to an Integrated Comprehensive Droplet Digital Detection. Flow-focusing microfluidic chip geometry producing encapsulated mycobacteria in droplets. Figure created on Biorender.com, accessed on 26 October 2021.

Detecting drug-resistant strains early in the infection will aid clinical decision making and shorten the time for optimal drug treatment. Sophisticated detection of resistant strains will also transform drug discovery and innovation within the laboratory. Researchers detected single-nucleotide polymorphisms between RIF-resistant *M. tuberculosis* isolates and susceptible isolates by combining a microfluidic chip with post-PCR high-resolution melting analysis (HRMA). The authors’ “Light Forge” microfluidic DNA melting-based TB test showed better performance of melting temperature differences compared to conventional Sanger sequencing, as well as a HRMA device on its own and phenotypic drug susceptibility testing [138]. Additionally, evidence shows that by incorporating open-chip microfluidics with padlock probe (PLP) ligation and rolling circle amplification (RCA), a two-hour assay is achievable for detecting an INH resistance caused by mutations in the gene (katG) in *M. tuberculosis.* The lab-on-a-disc platform utilised separate fluidic chambers for ligation and amplification steps, which provided temperature control [139]. Law et al. (2018) combined a lab-on-a-disk and recombinase polymerase amplification to fluorescently detect the pathogen with a sensitivity of 10^2^ colony-forming units per millilitre [140]. Drug-resistant strains to β-lactams were fluorescently detected using a droplet-based microfluidic device and a custom 3D particle counter (Figure 3). The microfluidic chip comprised separate input channels for bacteria, ampicillin and broth mixture, fluorocillin (a β-lactamase sensor), and oil to encapsulate single bacteria cells into droplets. Antibiotic-resistant clinical isolates could grow inside the droplets, detected by fluorescent microscopy [141]. 

Investigators are overcoming the challenge of genotyping drug-resistant strains of the pathogen directly from sputum. Researchers detected and genotyped RIF and INH resistance by creating a closed system composed of a microfluidic amplification microarray [142]. Likewise, the lab-on-a-film platform created by Kukhtin et al. (2020) integrated amplification, hybridisation, washing, and imaging. The authors reported *M. tuberculosis* detection in sputum as 43 CFU/mL; however, future work of this method includes sensitivity investigation [143].

More importantly, it is vital to consider the translation to commercialisation of microfluidic diagnostic devices. Suitability, such as user-friendliness, portability, and economic feasibility, should be addressed at the basic research level. Alternative methods to fabricate microfluidic devices are by printed circuit board (PCB) technology. A lab-on-a-printed circuit board (LoPCB) was integrated with a biosensor system able to detect INFy for diagnosing TB [144,145]. The simple assay holds promise for developing a handheld, fully automated device for TB diagnostics. On a successful road to commercialisation, a lab-on-a-chip assay—VereMTB—has undergone a pilot study. The lab-on-a-chip integrates PCR and microarray to detect the *M. tuberculosis* complex, RIF and INH resistance, and NTM in less than 3 h [146,147]. The pilot study states that the detection kit of the VereMTB system for 124 sputum samples had 97.0% sensitivity and 98.3% specificity for MTC complex detection, as well as high specificity and sensitivity for RIF and INH resistance detection. On the translational path to commercialisation, the device has shown the feasibility of efficiently detecting clinical specimens [148]. It can be concluded that microfluidic chips have been used in combination with PCR, biosensors, and microscopy techniques for detecting TB infection and resistant strains (Table 1).

### 3.2. Applications of Microfluidics for TB Drug Discovery 

Microfluidic technologies have been developed for portable and disposable TB diagnostics, but more recently there have been attempts to bridge these microfluidic techniques with conventional antibiotic drug discovery for TB. A microfluidic system in combination with microspheres (comprising *M. tuberculosis*, peripheral blood mononuclear cells, and type I collagen) was achieved for the pharmacokinetic modelling of antibiotics. After establishing the 3D granuloma microsphere model, the fluidic system was used to mimic the pharmacokinetics of that seen in vivo by altering various concentrations of RIF over time. They observed fluctuations in killing over time compared with fixed antibiotic concentrations [149]. This method establishes a close resemblance to the range of drug concentrations over time in the body when a patient is exposed to antibiotics due to the adsorption, distribution, metabolism, and excretion (ADME) properties of the drugs administered.

Interestingly, Aldridge et al. (2012) studied heterogeneity between mycobacterial cell growth rates. Using a microfluidic chamber with live-cell imaging, they measured the elongation rates of single cells and concluded that due to the unipolar manner of bacteria growth, this causes heterogeneity in elongation growth rate. They selected the microfluidic chamber as it advantageously allows single cells to grow in the shallow chamber with fresh nutrient media diffusing across the channel. This permitted the ideal imaging of five generations of bacteria for growth studies [150]. A subsequent study using this model compared growth parameters and treatment responses to RIF. The microfluidic device cultured and imaged green fluorescent *M. smegmatis* cells. RIF was dispersed into the mixing device by a syringe pump to detect RIF-tolerant and -susceptible cells. The authors concluded an association of RIF tolerance with elongated cell length and advanced growth pole age at birth [151]. Additionally, using Aldridge’s microfluidic model and time-lapse microscopy, additional research explores the mechanisms by which heterogeneity has an effect on drug action. The authors found that a single gene, *lamA,* permits asymmetrical growth in replicating mycobacterial cells. *M. tuberculosis* cells deficient in *lamA* were more susceptible to RIF than wild-type bacteria. The authors suggest that by targeting *lamA*, scientists could in the future reduce the diversity in mycobacterial populations and thus eliminate persistence [152]. 

Time-lapse microscopy combined with microfluidics is proving useful to study population heterogeneity in bacteria strains. A group who are investigating a microfluidic application for tuberculosis previously used a microfluidic chamber to monitor *M. smegmatis* growth dynamics in real time. The visualisation of chromosome and replisome tracking were studied using time-lapse microfluidic microscopy (TLMM) [153,154,155]. After studying chromosome organisation, the authors then showed changes in cell replication and morphology following the addition of novobiocin, nalidixic acid, and griselimycin which are all replication-altering drugs [155]. The ability to study single-cell growth dynamics and changes in the replication complex upon the addition of antimicrobials will aid finding drug mechanisms of action for future drug discovery. Individual cell analysis was demonstrated using a confocal laser-scanning microscope and a microfluidic device. This allowed the growth dynamics and antibiotic killing of fluorescently labelled *M. smegmatis* to be measured in real time [156]. Drug mechanisms of action can also be found by monitoring metabolic changes induced by antibiotics by microfluidics. Baron et al. (2020) incorporated a microfluidic platform and wavelength-modulated Raman spectroscopy to trap live mycobacteria and analyse them optically. They discovered that monitoring the metabolic changes over time of bacteria induced by INH could be used to study different stress conditions in the future [157].

In addition to the previously mentioned imaging/microfluidic approaches, microfluidic live-cell imaging was combined with time-lapse microscopy to investigate the antimicrobial activity of peptoids (oligo-N-substituted glycines). The study concluded that the investigative molecules’ mechanism of action disrupted the cell membrane shown by the increased rate of uptake of propidium iodide [158]. 

**Figure 4:**
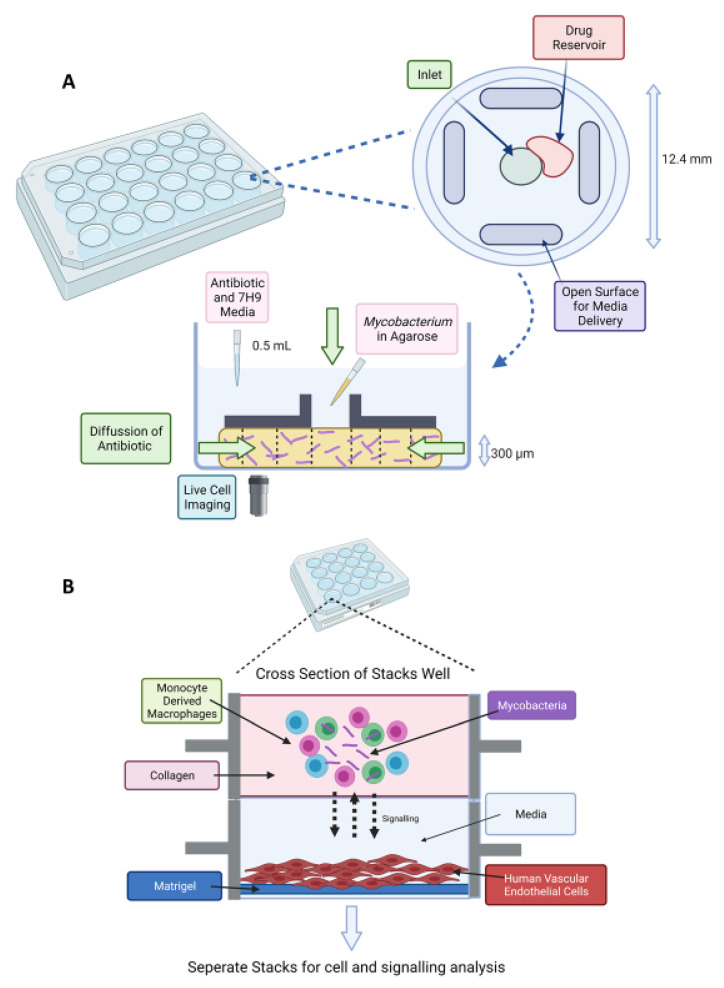
(**A**) Stacks microfluidic platform for analysing soluble signalling. The top layer contains the granuloma model in collagen and the bottom layer contains human vasculature endothelial cells on a Matrigel layer. Soluble factor signalling can be studied from the granuloma microenvironment [164]. (**B**) Schematic of DAC system. A 24-well plate fabricated from poly(methyl methacrylate) (PMMA) with a top view of an individual well (diameter of 12.4 mm and height of 12 mm). The disc-shaped channel (300 μm depth) is filled with agarose and mycobacteria. An inverted microscope is placed underneath for time-lapse imaging [159]. Created on BioRender.com, accessed on 26 October 2021.

Drug susceptibility testing utilising microfluidics has been attempted, which is faster than conventional approaches. *M. tuberculosis* has been immobilised in an agarose matrix and introduced to antibiotics which diffuse into the agarose on a microfluidic chip [159]. The agarose enables single cells that are being monitored by time-lapse imaging to remain stationary compared to liquid cultures. Minimal inhibitory concentrations (MIC) were determined by day 9 of experiments compared with weeks of conventional methods. Subsequent publications of this “Disc Agarose Channel (DAC)” system provided optimisation and validation (Figure 4A). The system was not sensitive to the initial inoculum effect, and MIC experiments with first- and second-line antimicrobials were achieved in 7 days [160]. Performance was then compared to conventional drug susceptibility testing when the microfluidic chip was optimised for commercial use and proved high agreement rate of 97.8% with a faster turnover time. A unique advantage of the DAC system is that it reduced TB leakage by a sealing film and locking lid, and serial dilution was not required, providing safety for laboratory researchers. 

Investigating the mechanism of antibiotic tolerance was demonstrated using wild-type and *msm2570::Tn* mutants of *M. smegmatis.* Single cells were studied using microfluidics and time-lapse microscopy and provided evidence that the mutant strain was more tolerant to INH compared to the wild-type strain. This current example is proof that microfluidics can achieve an improved understanding of resistance mechanisms of mycobacteria to antibiotics and aid the discovery of new antimicrobials [161].

The advancement of 3D models such as organ-on-a-chip has provided researchers the tools to study drug actions with more efficient in vitro models. This could reduce the use of animal models. For example, a lung-on-a-chip model was used to mimic the alveolar lung environment of early TB infection. A porous membrane creates an air–liquid interface of alveolar and vascular compartments and was then used to study the effect of pulmonary surfactant on mycobacteria infection of alveolar epithelial cells and macrophages. Utilising time-lapse microscopy, the model not only mimicked the host–pathogen interaction but also found that pulmonary surfactants had a protective role against TB, as shown by increased intracellular bacterial growth when cells lacked surfactants [162].This lung-on-a-chip model could aid drug discovery screening, which is more physiologically relevant. Previous studies have shown the utilisation of microfluidics for creating in vitro models of TB granulomas. “Stacks” is a previously cited microfluidic co-culture platform which enables extracellular signalling between different layers of cell types [163]. Building on this platform, an in vitro model was shown of an internal mycobacterial infection and its surrounding environment (Figure 4B). They used the model for soluble factor signalling studies, and further utilisation could explore the various immune signalling pathways of TB pathology [164]. Moreover, applications of engineered oxygen sensing in cultures could pave the way for controlling oxygen content when optimising new models of NRP TB. Measuring the concentration of oxygen in picodroplets has been demonstrated. Researchers successfully measured oxygen concentration against optical density (600 nm) of *Escherichia coli* and *Mycobacterium smegmatis* by utilising optical sensor nanoparticles. The nanoparticles had a phosphorescent indicator dye embedded in poly (styrene-blockvinylpyrrolidone) nanobeads and were easily integrated into a droplet device [165]. Monitoring analytes or conditions which influence bacterial growth is important in microbiology research and could be advanced by using microfluidic “stochastic confinement” droplets.

### 3.3. Future Application of Drug Screening in Microdroplets

The feasibility of using encapsulation technology to rapidly detect and enumerate individual microorganisms in patient samples has been demonstrated [166]. Effective control of TB transmission in vulnerable population groups is dependent on the rapid identification of the infectious agent and its drug susceptibility. *M. bovis* BCG and *M. smegmatis* were encapsulated in gel microdroplets, with a mean diameter of 25 μm, along with flow cytometry as a model system to investigate the efficacy of encapsulation and the detection of clonal growth by flow cytometry [167]. The characteristic slow growth of these microorganisms, as well as the small number found in most clinical samples, has made the direct detection of TB bacilli by biochemical and immunological methods difficult. Use of gel microdroplet encapsulation in combination with flow cytometry could reduce the time required to evaluate clinical samples and establish effective treatment regimens. 

One advantage of droplet microfluidics is the approach of “stochastic confinement” [168]. When single cells are confined in microdroplets of small volume, the loading is defined by Poisson statistics. When less than one bacterium is encapsulated per microdroplet, the resultant library of droplets is either singly occupied or empty. As detection time is proportional to the plug volume, then the random statistical probability of confinement effectively increases the cell density and subsequently reduces the time required for their detection. Using a microfluidic hybrid method, a variety of antibiotics were screened against a single bacterial sample. *E. coli* cells have been encapsulated in agarose monodisperse microparticles, approximately 30 μm diameter, using a flow-focusing microfluidic chip. Both the MIC for RIF and the sorting of spontaneous mutants by fluorescence-activated cell sorting (FACS) was demonstrated and characterised by DNA sequencing [169]. Building on this previous work, FACS screening of gel microdroplets has been shown, in which the bacterial pathogen *Staphylococcus aureus* is co-cultured with a recombinant host—*Saccharomyces cerevisiae* or *E. coli*, which are capable of secreting biocatalytic antibiotics and/or secondary metabolites from a metagenomic library [170]. The gel microdroplets (25 pL) are of a size compatible with conventional FACS instruments at 3000 droplets/second, allowing the proof-of-concept selection of antibiotic-secreting yeast from a vast excess of negative controls [170]. 

The frequency of resistance (FOR) of INH- and EMB-resistant mutants has been measured to be in the order of 1 × 10^−7^–1 × 10^−9^, respectively. Hence, to obtain a reasonable number of mutants (10–100 for DNA sequencing), at least 10^9^ bacteria would have to be screened. A label-free high-throughput method was previously reported for screening up to 1 × 10^9^ bacteria for AMR in water-in-oil picolitre-volume droplets (picodroplets); using Poisson statistics, the occupancy per picodroplet was 100 bacteria (*E. coli* HS151) [171]. From roughly 10 million picodroplets that were screened against fusidic acid, 103 droplets with drug-resistant hits were sorted. The recovered cells were grown on agar containing fusidic acid (10 μg/mL) and the mutant colonies submitted for DNA sequencing. The flexibility of alginate hydrogel beads (65 nL, 500 μm diameter) have the advantage of being able to be shuffled back and forth between the hydrophobic and hydrophilic phase. Schmitt et al. (2019) demonstrated the co-encapsulation of a library of *Lactococcus lactis* cells producing antimicrobial lanthipeptides with approximately 150 sensor strain cells, *Micrococcus flavus*, then back to the hydrophilic phase for the activation of lanthipeptide production, and back to the hydrophobic phase for incubation and to prevent lanthipeptide crosstalk between the microdroplets [172]. Finally, these nanolitre reactors (nLRs) were demulsified and stained with the fluorescent dye SYTO 9, and nLRs with no or only very little biomass, indicating the effective prevention of sensor strain growth, were isolated. Although this has yet to be carried out with mycobacteria, this technology holds considerable promise to screen antimicrobials against *M. tuberculosis* at the single cell level.

## 4. Discussion and Future Perspectives

Microfluidics is gradually transforming TB research. The application of fluidic devices in TB diagnostics, microbiological research, and drug discovery has proven the power of this technique in improving throughput and sensitivity and reducing dependence on animal models. Combining microfluidics with diagnostics and detection is ahead of the field compared to drug discovery. Chips were used in combination with PCR, biosensors, and microscopy techniques for detecting TB infection and resistant strains. We can now easily observe mycobacterial cell morphology when coupling microfluidics with imaging techniques which could aid our understanding of mycobacteria heterogeneity. Overall, it was found that the materials and methods used to fabricate microfluidic chips for TB investigation were biocompatible and cheap, making them suitable for long culture times and use in low-resource settings. 

Droplet fluidics is still rapidly evolving and will continue to grow with the success of its applications. There is a potential to incorporate novel droplet systems for studying biological processes for TB, such as cultivation vessels, investigating environmental stimuli, and the killing activity of antibacterials due to the ability of droplet sorting, injection, incubation, and mixing [173]. Droplets allow for the compartmentalisation of single cells from high-density cultures and the manipulation of cell environments. Thereby, droplets could prevent competition for nutrients or space among mycobacteria, allowing the slow-growing species to proliferate. From this literature review, it is noted that the use of droplet fluidics has not been fully applied for TB. To our knowledge, three studies have shown droplet applications in mycobacteria including detection [133], differentiation of species [132], and detecting resistant strains to antibiotics [141]. As evidenced by the ground-breaking research within the field of microfluidics and its use for drug discovery, undoubtedly there is an increased outlook of standardised microfluidic devices to test antimicrobials against mycobacteria and to discover their mechanisms of action. As other studies have investigated the drug screening of different microorganisms in microdroplets, there is scope and a gap in the literature for combining droplet fluidic technology at the picolitre scale with TB drug discovery. Microfluidic models are yet to replicate the NRP state of the mycobacteria and carry out antibiotic susceptibility testing in a standardised and high-throughput manner. There is an opportunity to create a new, faster, and more sensitive method of antimicrobial susceptibility testing which is more clinically relevant to the NRP state of the bacterium.

Future research should focus on translating these laboratory platforms into commercial application for industry and clinical practice. Commercial products of single-cell droplet platforms have been successful, such as inDrop, Drop-seq, and 10× Genomics [173]. Interestingly, as previously mentioned, a droplet system is on the path to commercialisation for TB resistance detection [148]. Challenges for the commercialisation of these novel platforms include translation, user-friendliness, portability, and economic feasibility. Interdisciplinary collaboration has facilitated these advancements, usually involving biologists and engineers and their respective stakeholders. Challenges to overcome in this multidisciplinary field include the scale-up of testing and parallelisation for industry usage. Furthermore, transfer of “know-how” between designers and end users is imperative. With the miniaturisation of biological assays, more robust data points are obtainable and may need bioinformatic expertise and sophisticated computational tools. Machine learning has the ability to learn from high-throughput data. It has previously been used in TB chest X-ray diagnosis, which helps to prevent the overdiagnosis or underdiagnosis of TB made by variations in X-rays [174]. Converging microfluidic analysis with machine learning could provide high-throughput accuracy and prediction models in the field of TB [175]. Machine learning algorithms made from large datasets obtained from microfluidic chip arrays will possibly predict antimicrobial resistance to tuberculosis. This would aid empirical treatment to find the right treatment option for the patient at the right time [176,177]. This provides an opportunity for paving a “precision medicine”-based therapy option when deciding what drugs to give to patients. With the rapid detection of resistant clinical strains in hospital environments, patients could receive the correct choice of antimicrobial at the correct time, eliminating the infection faster and reducing antimicrobial resistance. 

It is encouraging to highlight the breadth of research utilising microfluidics and multidisciplinary collaboration for furthering our understanding of TB, its diagnosis, and how best to manage it.

## Figures and Tables

**Table 1:** Summary of current microfluidic applications for diagnostics and detection of *M. tuberculosis*.

Sample	Devices Applied	Applications
Direct bodily fluids Detection of clinical isolates, model organisms, attenuated strains Detection of drug resistant strains	Microfluidic open chips Lab-on-a-chip Droplet Fluidics	Polymerase Chain Reaction Loop-mediated isothermal amplification (LAMP) Recombinase polymerase amplification (RPA) Electrochemical Biosensing Fluorescence detection Surface-enhanced Raman spectroscopy Microscopy

## Data Availability

Not applicable.
